# Remembering Dr. Ronald Weinstein

**DOI:** 10.1016/j.jpi.2022.100015

**Published:** 2022-02-09

**Authors:** Keith J. Kaplan

**Affiliations:** Alexian Brothers Medical Center, Elk Grove Village, IL, USA

To the editor,

I first met Ron Weinstein at a United States and Canadian Academy of Pathology (USCAP) meeting in the early 90’s as a resident. Being interested in basic computing at that time for pathology databases, images, and references, I sought to introduce myself to him. The World Wide Web was still in relative infancy at that time. Ron had already established himself as an innovator in telepathology, a term he would coin at a USCAP meeting several years prior. The idea of moving images, not slides, for collaboration, consultation, education, and research resonated with me. Walter Reed Army Medical Center, where I was completing my residency, had one of his first telepathology systems. By the time I saw it on the floor of the lab behind some boxes under a counter, it had not seen use for many years. He was very gracious with his time and showed me several papers he was in the process of reading, what problems he found with them, drafts of other manuscripts, and notes concerning practice and business models for telepathology. He was going to use communication channels that were already in use in other sectors, such as the financial industry, for medicine, and specifically pathology.

When the Army sought to develop its own telepathology program with the Armed Forces Institute of Pathology, I again called on Ron for his mentorship and advice. His virtual mentorship from Arizona to DC proved invaluable. His vision of providing pathology services to remote and underserved areas resonated with me in the military’s goal “to provide subspecialty care as far forward as possible”. A few years later robotic telepathology systems were installed in Iraq, Korea, and Germany as well as deployed throughout the United States. While many doubted this could be done, Ron was always enthusiastic and supportive and there to lend moral support.

While Ron was president of the American Telemedicine Association (ATA), I came to know how passionate he was while serving as a member of the Special Interest Group for Telepathology at the ATA. He worked tirelessly to ensure the technologies were appropriate for clinical use and to seek appropriate reimbursement from insurers for their use. While telemedicine may have been a foreign word for many prior to 2020, Ron was on the leading edge, insuring safe and effective use and coverage for payment for such services. On a personal level, he would share stories with me about his time in the Air Force, his time as the youngest chairman of pathology while at Rush Medical College and we would compare notes on our experiences in Chicago.

As robotic technologies started to be replaced by whole slide imaging, Ron again was on the leading edge of this using lens array technology with optical engineers from the University of Arizona, adding to Ron’s patent portfolio. While those products are no longer commercially available, his vision helped drive the need for high resolution whole slide images, that today are approved for clinical use for primary diagnosis. His first remembrances of black and white images from Logan Airport to Massachusetts General Hospital (MGH) had led to microscopic lenses capturing an entire glass slide all the way across the country.

**Image 1:**
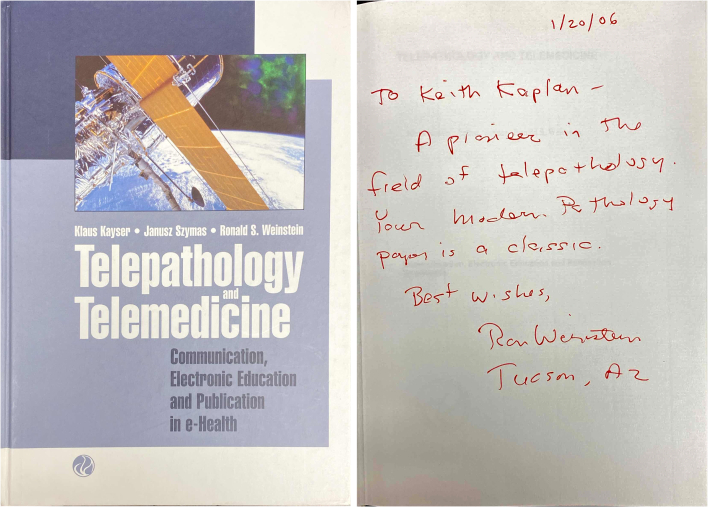
Copy of Dr. Ron Weinstein's signed book on telepathology.

About 16 years ago, this month, I visited Tucson for a College of American Pathologists (CAP) committee meeting. I really don't recall the committee, perhaps instrumentation or standards. But I do recall going to Ron's office with a copy of his latest book in hand, anxious to have him put his name on it and spend a few minutes with me. An afternoon later with a tour of his commercial operations and a signed copy of the book (Image 1), I returned to the CAP hotel.

Several years later Ron and I gave a talk at a national meeting. We planned out who would cover what topics. I started by saying I would cover the history of telepathology. Politely, Ron stopped me and said he should cover the history of telepathology; he lived it. He was right, from the early days of moving black and white images from Logan Airport to MGH, to the first demonstrations between El Paso, Texas and Washington, District of Columbia, and many more, he did it.

The Arizona Telemedicine Program, which Ron founded, stands a living legacy to Ron’s tireless work to extend and expand healthcare services which now links 160 sites in 70 communities and has provided care in 61 subspecialties of medicine.

I last spoke with Ron last year; he was still as excited as ever about his telemedicine program and the great progress they had made across the state. He talked about further expansion and the need for more providers to provide more services. He was still reviewing pathology images, now on a much larger, higher resolution monitor than he ever thought was possible. He spoke about what the pathology community needed to do to adopt digital pathology and how the lack of pathologists in the future will make digital pathology a necessity rather than a “nice to have”. He suggested we give an update on telemedicine and telepathology and talk about our ongoing work and showcase the latest technologies, when in-person meetings resumed.

As the father of telepathology, Ron helped spawn the field of pathology informatics, mentored hundreds of pathologists to develop and promote the use of technology within our specialty and has touched the lives of millions of patients and their families.

I am honored to have known Ron as a mentor and friend and we as a specialty and a community for having him amongst us for so long, active and always encouraging us to think about how to use technology to improve our lives and the lives of our patients, are better because of him.

